# Fabrication of Biocompatible, Vibrational Magnetoelastic Materials for Controlling Cellular Adhesion

**DOI:** 10.3390/bios2010057

**Published:** 2012-02-13

**Authors:** Hal R. Holmes, Ee Lim Tan, Keat Ghee Ong, Rupak M. Rajachar

**Affiliations:** Department of Biomedical Engineering, Michigan Technological University, Houghton, MI 49931, USA; E-Mails: hrholmes@mtu.edu (H.R.H.); eltan@mtu.edu (E.L.T.); kgong@mtu.edu (K.G.O.)

**Keywords:** magnetoelastic (ME) material, Parylene-C, biocompatibility, plasma etching, cellular adhesion

## Abstract

This paper describes the functionalization of magnetoelastic (ME) materials with Parylene-C coating to improve the surface reactivity to cellular response. Previous study has demonstrated that vibrating ME materials were capable of modulating cellular adhesion when activated by an externally applied AC magnetic field. However, since ME materials are not inherently biocompatible, surface modifications are needed for their implementation in biological settings. Here, the long-term stability of the ME material in an aqueous and biological environment is achieved by chemical-vapor deposition of a conformal Parylene-C layer, and further functionalized by methods of oxygen plasma etching and protein adsorption. *In vitro* cytotoxicity measurement and characterization of the vibrational behavior of the ME materials showed that Parylene-C coatings of 10 µm or greater could prevent hydrolytic degradation without sacrificing the vibrational behavior of the ME material. This work allows for long-term durability and functionality of ME materials in an aqueous and biological environment and makes the potential use of this technology in monitoring and modulating cellular behavior at the surface of implantable devices feasible.

## 1. Introduction

The host response to an implanted biomaterial, particularly the excessive accumulation of extracellular matrix components (ECM), is a key concern to the ultimate success of many biomedical implants [1,2,3,4]. Under normal circumstances, the host response can potentially be regenerative in nature, recapitulating lost or injured cells, as well as fibrotic, wherein damaged parenchymal tissue is replaced by connective tissue [2]. However, in cases of chronic or sustained inflammation (*i.e*., long-term transcutaneous resident implants), the fibrotic phase can be pathologic, leading to uncontrolled remodeling of the ECM and the long-term destruction of healthy tissue-implant architecture [2,3]. Surface properties of an implant are known to have a dramatic effect on the staging of the host response during the first few weeks in service [1]. For this reason a significant research effort has been aimed at developing surface treatments to control, establish, and maintain application appropriate, stable implant-tissue interfaces.

For each specific intended use, a biomaterial tissue interface must establish and maintain its chemical and physical properties and function by directing or modulating the acute and ultimately chronic host response in order to achieve optimal performance. The material must provide a tailored environment to ensure both sustained tissue structure and function but also if possible to direct desired cell behavior such as orientation and migration to ensure that the appropriate cells migrate to and/or adhere to the implant. Approaches involving pre-implantation design and preparation of material surfaces, transient surface transformation, *in situ* delivery of surface established architecture, or molecular release can significantly affect the host response for a brief period immediately following implantation, but are unable to provide real-time *in situ* control and feedback at an implant tissue interface. We have recently reported on a potentially novel approach for the therapeutic treatment of pathological fibrosis that can be activated post-implantation and used to remotely modulate and monitor cell adhesion *in vitro* [5]. The approach is based on magnetoelastic (ME) materials, traditionally used as biosensors for monitoring physical parameters such as temperature [6,7], pressure [7,8,9,10], and viscosity and flow velocity of liquids [10,11,12,13]. These materials function by converting magnetic energy into mechanical energy via cyclic elastic deformation (vibrations) in the presence of an external AC magnetic field. Overall deformation is a product of the initial length of the ME material and final strain amplitude [6,7,8,9,10,11,12,13,14,15,16,17]. The elastic recovery of the material also generates a secondary magnetic field that is proportional to the applied mass at the substrate surface [17]. This secondary field can be used to monitor changes (*i.e.*, cell and protein adhesion, and local consolidation of the tissue at the material interface) occurring at the substrate surface *in situ*. This novel application of ME materials represents a non-invasive means to locally modulate the host response *in situ* coupled with a unique feedback system to monitor the soft-tissue implant interface in real-time [5]. However, ME materials alone do not possess sufficient barrier properties (*i.e.*, corrosion resistance) to function long-term *in vivo* [5]*.* Therefore, the aim of this work is to develop a thin film coating that can sustain long term stability for controlled cellular adhesion at the soft tissue-implant interface.

**Figure 1:**
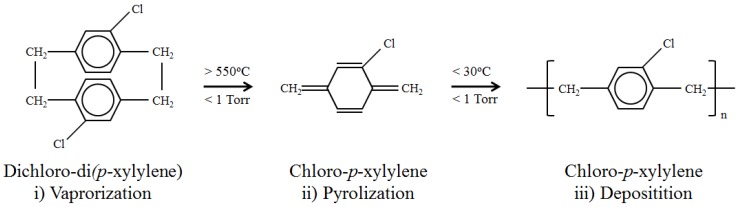
The Chemical Vapor Deposition process of Parylene-C. The dimer dichloro-di(*p*-xylylene) is cleaved into two chloro-*p-*xylylene monomer units and undergoes sublimation at temperatures about 550 °C and pressures less than 1 torr. The monomer units are then deposited onto a surface where they spontaneously polymerize at room temperature.

For this study, we investigate the capabilities of poly-(chloro-*p*-xylylene), more commonly known as Parylene-C, as the biocompatible coating for magnetoelastic materials. Parylene-C is designated by the United States Pharmacopeia as a Class IV polymer, the highest level of biocompatibility for polymers, making it permissible for long-term implantation [18]. Films made from chemical vapor deposited Parylene-C possess many desirable properties for medical applications, including chemical inertness and resistance to biological degradation [19,20], and have already seen use in a variety of biomedical devices such as cardiovascular implants, wireless neural interfaces, and catheters [18,20]. Additionally, Parylene-C is often chosen for medical use due to its deposition process (Figure 1) [18,19,20,21]. Advantages of this deposition process include excellent adhesion, a solvent-free environment, and high accuracy and control over film thickness [21,22]. Ultimately this process results in coatings that are highly resistant to hydrolytic degradation and stable in a biological environment [18,19,21]. 

The relative inertness of Parylene-C provides for surfaces that are resistant to cell and protein interaction. However, in the case of transcutaneous implants [23] further control over the progression and location of host response activity is often required. To address this issue, simple processes, such as air plasma etching [19] and soft lithographic patterning [21], that are capable of providing Parylene-C surfaces with additional functionality have already been developed. Combining the ability to customize Parylene-C surface properties with the long-term therapeutic and monitoring capabilities of ME materials could provide for a robust, highly controllable implant coating that can be easily modified for specific applications.

The first goal of this work is to investigate the ability of Parylene-C coatings to allow for long-term use of ME materials *in vitro* and *in vivo* without compromising the magnetostrictive properties that provide for the functionality of ME materials. The second is to understand if Parylene-C coatings can be functionalized or modified to provide further control of cell adhesion. These objectives will be accomplished by characterizing the effect of a Parylene-C coating on the mechanical properties of the ME material, determining if Parylene-C encapsulation of the ME material provides sufficient barrier properties to prevent degradation, and investigating methods of functionalizing Parylene-C coatings for cell attachment and the respective responses to mechanical loading by ME materials.

## 2. Materials and Methods

### 2.1. ME Material Preparation and Parylene-C Coating

In preparation for Parylene-C coating, mechanically sheared ME materials (Metglas 2826MB-Fe_40_Ni_38_Mo_4_B_18_-Metglas Inc.) were cleaned and heat-treated (120 °C for 2 h) to reduce the internal stress and improve their magnetic properties. Parylene-C was then coated onto the ME materials using a Parylene deposition system (PDS 2010 LABCOTER^TM^ 2) following the manufacturer’s recommended protocol. Prior to implantation and *in vitro* culture, Parylene-C-coated ME materials were sterilized with ethylene oxide (EtO) gas. Final ME sample dimensions were 5 mm × 12.8 mm × 26 µm for all experiments.

### 2.2. Oxygen Plasma Etching

Parylene-C (thickness of 10 µm) coated ME materials (*n* = 6 per group) were weighed and characterized by resonant frequency as previously described [17]. ME materials were then etched with oxygen plasma (200 mTorr) using a March Jupiter II RIE system for 0.5, 1, 3, or 5 min. ME materials were again weighed and characterized by resonant frequency following etching completion.

### 2.3. Surface Characterization

Surface topography measurements were made with a Nanoscope E (Digital Instruments) AFM system using constant deflection mode with a micro-fabricated silicon nitride cantilever in air. Images were processed using Digital Instruments AFM software to calculate root means squared (RMS or *Rq*) roughness, defined as the standard deviation of elevation with respect to the mean of a scanned measured area (5 µm^2^) taken over 9 sections per group. Hydrophobicity was characterized using sessile drop contact angle analysis of deionized-H_2_O with a minimum of 9 measurements per sample.

### 2.4. Effect of Coating on the ME Vibration

**Figure 2:**
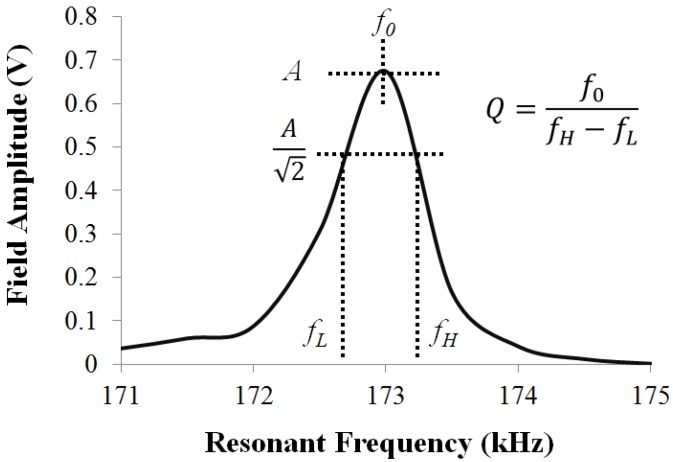
A typical resonance of a ME material, where the resonance behavior is characterized by the resonant frequency (*f_0_*), resonant amplitude (*A*), and resonant quality (*Q*).

To investigate the effect of Parylene-C coating on the vibration of the ME material, the resonance behavior of the material was examined. As illustrated in Figure 2, useful characteristics of the ME resonance include: (i) the resonant frequency (*f_0_*), which is the frequency when the vibration of the material is at its highest; (ii) the resonant amplitude (*S*), which is the highest vibration amplitude; (iii) the resonant quality, which characterizes the spread of the resonant peak. The resonance of the ME material was captured using previously established approach [17]. Briefly, the ME materials were mechanically vibrated by exposing them to a selected frequency range of AC excitation field (160–165 kHz). In this study, ME materials coated with 5, 10, and 20 µm (*n* = 10 for each thickness) of Parylene-C were selected for investigation.

### 2.5. Protein Adsorption

ME materials (*n* = 3 per group) coated with Parylene-C (thickness of 10 µm) were incubated in a solution of fibronectin (9.6 μg/mL in Hank’s Balanced Salt Solution, HBBS) for 45 min at room temperature, and vitronectin (0.48 μg/mL in HBSS) for 2 h at 37 °C. Resulting surface coverage of ME materials for fibronection and vitronection were 2 and 0.1 μg/cm^2^, respectively. After rinsing the samples with HBSS, L929 fibroblasts were then seeded onto protein coated ME materials at a density 2 × 10^4^ cells/cm^2^ and stained with a live fluorescence assay after two days of incubation at 37 °C and 5% CO_2_. 

### 2.6. Cell Culture and Adhesion

For all experiments involving cell culture, L929 fibroblasts (ATCC Catalog No. CCCL-1) were maintained in standard culture medium composed of 10% FBS and 0.5% ATCC Penicillin/Streptomycin in Dulbecco’s Modified Eagles Medium (Cellgro Catalog No. 10-017-CM). Chamber slides (Lab-Tek^TM^-Chamber Slide^TM^ system) were used to house ME materials and subsequent culture. Live/Dead assays were performed using Calcien-AM (Fluka 11783) and Ethidium Bromide (Sigma-Aldrich), respectively. Images were taken for cytotoxicity on inverted Axiovert 200 M Zeiss microscope and for adhesion on upright Olympus BX51 microscope. For vibration loading experiments, ME vibrations (Frequency 160–165 kHz) were applied to experimental set for 1 h prior to staining using previously developed *in vitro* ME vibrational loading system [5].

Parylene-C (thickness of 10 µm) coated ME materials (*n* = 3 per group) etched with oxygen plasma (0.5, 1, 3, or 5 min) were cultured with L929 fibroblasts. Cells were directly seeded on the ME materials at a density of 2 × 10^4^ cells/cm^2^. Following incubation for 48 h at 37 °C and 5% CO_2_, live/dead assays were performed using Calcien-AM (Fluka 11783) and images were obtained with an upright Olympus BX51 microscope.

### 2.7. *In Vivo* Evaluation

In this study, a bi-lateral subcutaneous implantation model was used as a simple reproducible means to establish that the biocompatible Parylene-C coating of ME materials was viable under a dynamic *in vivo* host response [24,25,26]. Parylene-C coated (10 µm) and 0.5 min plasma-treated ME materials were subcutaneously implanted into the dorsal side of three age-matched male BALB/c mice (Harlan Laboratories) for 30 days using previously established implantation techniques [24]. Animals were housed and used in specific-pathogen-free facilities according to a protocol approved by the Institutional Animal Care Use Committee at Michigan Technological University. At the end of each implantation, the ME materials and surrounding tissue capsule were explanted and qualitatively assessed.

### 2.8. Statistical Analysis

All experiments were performed in triplicate, unless otherwise stated. Statistical analyses were made using standard student’s t-test or ANOVA (JMP^®^ software). Data is expressed as the mean ±S.E.M, and p-values less than 0.05 (p < 0.05) were considered significant.

## 3. Results and Discussion

### 3.1. Secondary Field Characterization

**Figure 3:**
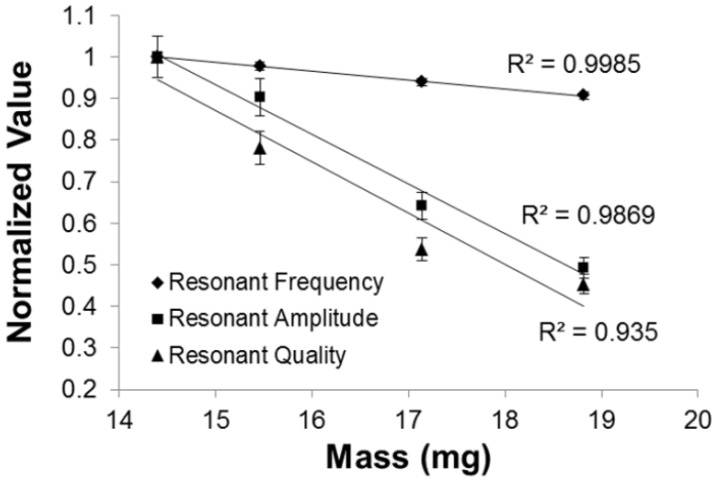
Additional mass loading as a result of Parylene-C coating reduced the resonant frequency, resonant amplitude, and resonant quality. Note that each resonant behavior exhibited standard deviation of less that 5%.

The additional mass loading upon deposition of Parylene-C coating is shown in Table 1. The effect of mass loading as a result of an additional Parylene-C coating on the resonance behavior of the ME material is shown in Figure 3. The application of Parylene-C coatings to ME materials dampened the vibration, and hence the secondary field response. When the Parylene-C coating thickness was increased from 0 to 20 µm, the additional mass loading reduced the maximum secondary field of ME material, *A*, from 0.67 to 0.33 V, a reduction of 50% on the resonant frequency. This observation is consistent with previous studies where additional weight on ME material would subsequently increase the resistance of ME vibration, hence, lowering the resonant frequency of the material. In addition, the resonant frequency and quality were reduced as a function of addition mass loading. While there was a reduction in resonant amplitude and quality, the ME materials were still able to retain their magnetostrictive properties when subjected to Parylene-C coating. The conformity of this trend also shows that the Parylene-C coating has stably integrated with the dynamic ME material substrate.

**Table 1:** The additional mass loading applied to ME material upon deposition of Parylene-C.

Parylene-C Thickness (µm)	Mass Loading (mg)
0	14.40
5	15.46
10	17.14
20	18.82

### 3.2. Parylene-C Functionalization via Oxygen Plasma Etching

**Figure 4:**
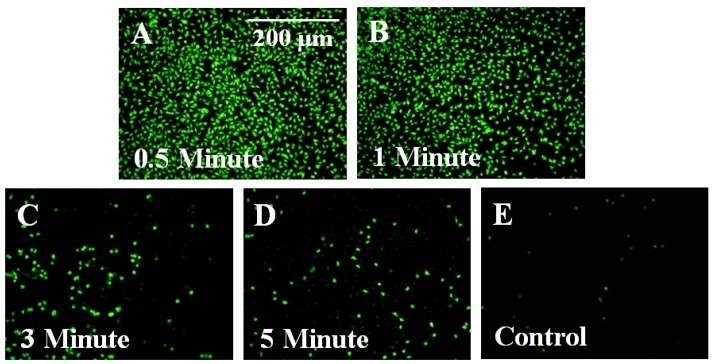
Treatment with oxygen plasma was shown to improve cellular adhesion on Parylene-C coated ME material. Fluorescent images for 0.5 and 1 min plasma-treated samples (**A** & **B**) revealed greater levels of fibroblast adhesion than samples that were plasma-treated for 3 and 5 min (**C** & **D**). (**E**) Untreated Parylene-C controls showed little or no evidence of cell adhesion.

Plasma etching was shown to affect Parylene-C coating by opening and removing the benzene ring following the sequential formation of a hydroxyl then peroxy radical at the ethyl carbon site [18]. Through this processing technique, only the outer most layers of the film will possess modified surface properties [21,27]. Here, Parylene-C coated ME materials treated with different duration of plasma etching (0.5, 1, 3, and 5 min) were used to evaluate the cell reactivity to plasma-etched surface. As shown in Figure 4, fluorescent images of cells cultured on Parylene-C coated ME materials demonstrated that short plasma treatments (0.5 and 1 min) could improve the cellular adhesion.

**Figure 5:**
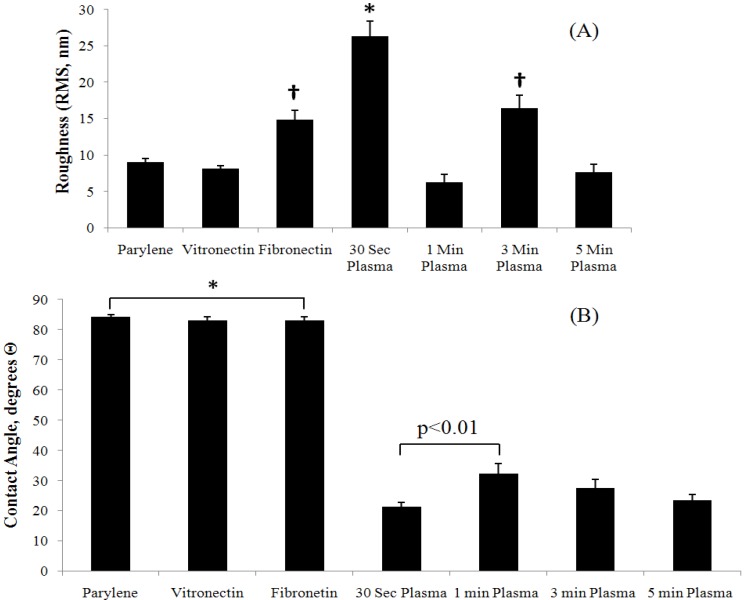
Surface properties characterization of plasma-treated and Parylene-C coated ME materials. (**A**) Surface roughness and (**B**) hydrophobicity analyses showed treatment methods involving plasma etching dramatically changed the surface character of Parylene-C while protein adsorption had relatively little effect on these specific surface properties. Statistically significant differences (p<0.01) between groups are indicated (*, †).

Changes in cellular adhesion may be attributed to changes in surface properties (surface roughness and hydrophobicity) resulting from oxygen plasma treatment. As indicated in Figure 5, significantly (p < 0.01) different roughness for 0.5 and 3 min plasma-treated samples (Figure 5(A)), and significantly (p < 0.01) different hydrophobicity for all plasma-treated samples (Figure 5(B)) were evident when compared to untreated Parylene-C. However, it is interesting that similar levels of cellular adhesion occurred on samples etched for 0.5 and 1 min despite having significantly different surface roughness and hydrophobicity. Additionally, lower levels of cellular adhesion occurred on 3 and 5 min samples that possessed properties more similar to samples treated for 1 min. This result shows that plasma etching may also affect surface properties other than those directly investigated in this study, such as stiffness.

**Figure 6:**
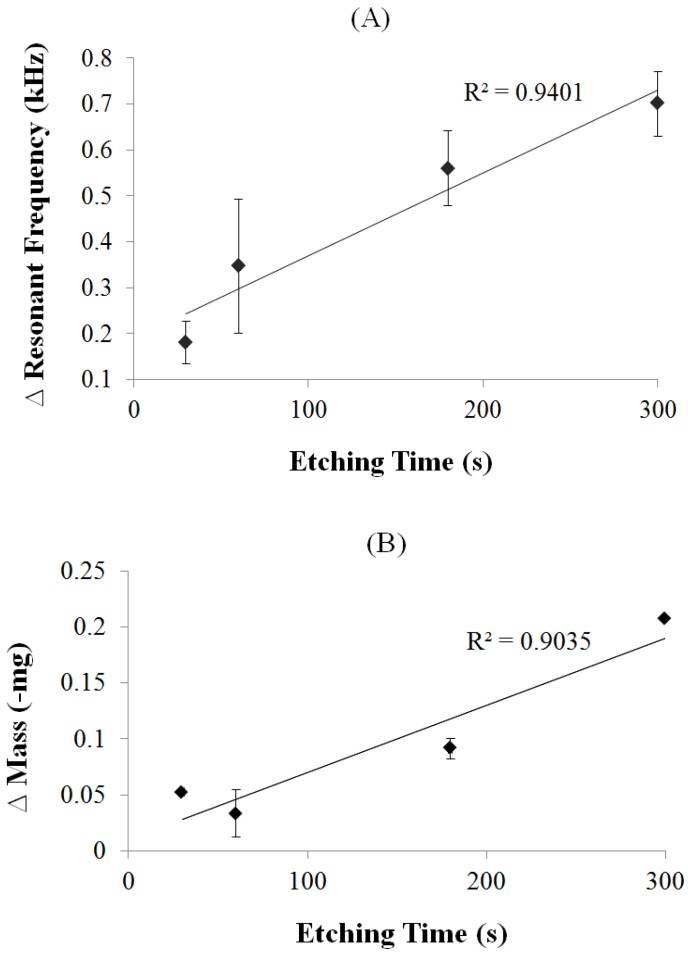
The correlation of plasma treatment to ME material’s resonant frequency, and mass change. Resulting resonant frequency (**A**) was inversely proportional to mass changes (**B**) as a result of plasma etching with a strong linear trend.

Using the ME material to monitor the effect of plasma etching on Parylene-C coated ME materials resulted in a trend of frequency increasing proportionally to etching time (*R^2^* = 0.9401, Figure 6(A)). This trend is consistent with the previously described effect of plasma etching (the removal of benzene rings corresponds to an overall change in mass), which is also consistent with the previous finding where the increase of mass decreases the resonant frequency and *vice versa*. The relationship of etching time and mass change is shown in Figure 6(B). As demonstrated, oxygen plasma etching technique was capable of controlling the coating mass, thus the resonant frequency, by simply altering the etching time.

### 3.3. Control of Cellular Adhesion on Functionalized Parylene-C Surfaces

**Figure 7:**
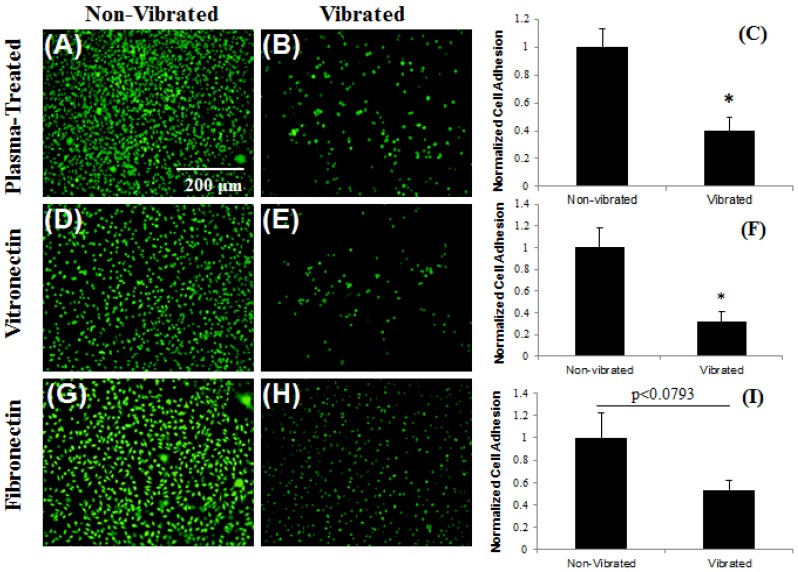
ME vibrations control cellular adhesion on functionalized (plasma-treated) Parylene-C surfaces. L929 fibroblasts cultured on Parylene-C coated ME materials plasma-treated for 0.5 mi (**A**–**C**) or adsorbed with vitronectin (**D**–**F**) showed significant reductions in cellular adhesion when subjected to ME vibrations. Cells cultured on Parylene-C adsorbed with fibronectin showed changes in cell morphology when subjected to ME vibrations, but significant detachment was not observed (**G**–**I**). Statistically significant differences (p<0.01) between groups are indicated (*).

The application of ME vibrations to fibroblasts cultured on functionalized Parylene-C coatings resulted in significant reductions (p < 0.01) in cell adhesion for samples adsorbed with vitronectin and plasma-treated for 0.5 min (Figure 7(A–F)). Qualitatively, noticeable changes in cell morphology (diminished cell spreading and increased roundness) were observed on samples absorbed with fibronectin, however there was no significant detachment (p < 0.0793) (Figure 7(G–I)). This outcome demonstrated the efficacy of working ME sensors in conjunction with a Parylene-C coating to modulate cell adhesion. The result also demonstrated the mechanical stability of the Parylene-C coating layer in a cell culture environment for 2 days (additional experiments showed the mechanical stability of the Parylene-C coating for a minimum of 6 months under culture conditions). Furthermore, this result showed that different functionalization techniques of Parylene-C coatings could be used to provide levels of control over the resulting cellular response to ME mechanical loading.

### 3.4. *In Vivo* Stability

**Figure 8:**
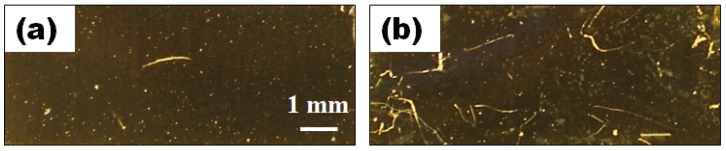
Parylene-C maintains ME material integrity *in vivo*. Images of ME material before (**a**) and after (**b**) implantation show no observable traces of corrosion after 30 days of subcutaneous implantation in BALB/c mice.

The application of 10 µm and 0.5 min plasma treated Parylene-C coating on ME material was shown to provide for material stability when subjected to a healthy mammalian host response. Qualitative assessment showed no signs of corrosion on explanted ME material after 30 days implantation (Figure 8). Furthermore, mice did not show any visible systemic effects to implants, exhibiting normal grooming and dietary behavior throughout the implantation period. This result demonstrated the feasibility and durability of plasma-treated and Parylene-C coated ME material within a dynamic biologic environment. Subsequent animal studies will be able to use this approach to explore *in situ* responses to local vibrational therapy where material stability inside a physiological environment is crucial to ensure long-term functionality of this technology.

## 4. Conclusions

The results of this study present *in vitro* and *in vivo* evidence that Parylene-C coatings effectively provide long-term stability of ME materials. Additionally, the mechanical character of ME materials is not eliminated as a result of this coating procedure, preserving the ability of the material to remotely modulate cell adhesion. Furthermore, treatment methods, including protein adsorption and oxygen plasma etching, can be employed to functionalize Parylene-C surfaces to create a robust system that allows for heightened control of cellular adhesion at the substrate surface. Lastly, Parylene-C coatings allow for the long-term use of ME materials in dynamic contact with a biologic environment. This coating system could become a novel approach for preventing interfacial fibrosis in transcutaneous devices by providing a means to promote stable integration during the entire service-life of the implant.
